# Determinants of Resting Oxidative Stress in Middle-Aged and Elderly Men and Women: WASEDA'S Health Study

**DOI:** 10.1155/2021/5566880

**Published:** 2021-06-07

**Authors:** Takuji Kawamura, Kumpei Tanisawa, Ryoko Kawakami, Chiyoko Usui, Tomoko Ito, Hiroki Tabata, Nobuhiro Nakamura, Sayaka Kurosawa, Wonjun Choi, Sihui Ma, Zsolt Radak, Susumu S. Sawada, Katsuhiko Suzuki, Kaori Ishii, Shizuo Sakamoto, Koichiro Oka, Mitsuru Higuchi, Isao Muraoka

**Affiliations:** ^1^Waseda Institute for Sport Sciences, 2-579-15 Mikajima, Tokorozawa, Saitama 359-1192, Japan; ^2^Faculty of Sport Sciences, Waseda University, 2-579-15 Mikajima, Tokorozawa, Saitama 359-1192, Japan; ^3^Sportology Center, Juntendo University Graduate School of Medicine, 2-15-8 Hongo, Bunkyo-ku, Tokyo, Japan; ^4^Institute of Advanced Active Aging Research, Waseda University, 2-579-15 Mikajima, Tokorozawa, Saitama 359-1192, Japan; ^5^Graduate School of Sport Sciences, Waseda University, 2-579-15 Mikajima, Tokorozawa, Saitama 359-1192, Japan; ^6^Research Fellow of Japan Society for the Promotion of Sciences, 5-3-1, Kojimachi, Chiyoda-ku, Tokyo 102-0083, Japan; ^7^Research Center for Molecular Exercise Science, University of Physical Education, Alkotas Str. 44, Budapest 1123, Hungary

## Abstract

Previous studies have not investigated the determinants of resting oxidative stress, including physical fitness, as it relates to redox regulation. The present study therefore was aimed at identifying lifestyle and biological factors that determine resting oxidative stress, including objectively measured physical fitness. In 873 middle-aged and elderly men and women, age and anthropometric parameters, lifestyle-related parameters, medication and supplementation status, physical fitness, biochemical parameters, and nutritional intake status, as well as three plasma oxidative stress markers: protein carbonyl (PC), F_2_-isoprostane (F_2_-IsoP), and thiobarbituric acid reactive substances (TBARS), were surveyed and measured. The determinants of PC, F_2_-IsoP, and TBARS in all participants were investigated using stepwise multiple regression analysis. In PC, age (*β* = −0.11, *P* = 0.002), leg extension power (*β* = −0.12, *P* = 0.008), BMI (*β* = 0.12, *P* = 0.004), and HDL-C (*β* = 0.08, *P* = 0.040) were included in the regression model (adjusted *R*^2^ = 0.018). In the F_2_-IsoP, smoking status (*β* = 0.07, *P* = 0.060), BMI (*β* = 0.07, *P* = 0.054), and HbA1c (*β* = −0.06, *P* = 0.089) were included in the regression model (adjusted *R*^2^ = 0.006). In TBARS, glucose (*β* = 0.18, *P* < 0.001), CRF (*β* = 0.16, *P* < 0.001), age (*β* = 0.15, *P* < 0.001), TG (*β* = 0.11, *P* = 0.001), antioxidant supplementation (*β* = 0.10, *P* = 0.002), and HbA1c (*β* = −0.13, *P* = 0.004) were included in the regression model (adjusted *R*^2^ = 0.071). In conclusion, the present study showed that age, anthropometric index, lifestyle-related parameters, medication and supplementation status, objectively measured physical fitness, biochemical parameters, and nutritional intake status explain less than 10% of oxidative stress at rest.

## 1. Introduction

Humans and many other organisms take in oxygen, which is present in approximately 21% of the atmosphere, and use it to survive. However, during normal metabolic processes that utilise oxygen for the synthesis of ATP, some oxygen is converted into reactive oxygen species (ROS) such as superoxide, hydrogen peroxide, and hydroxyl radicals. Moderate ROS play an essential role in maintaining physiological homeostasis, including cellular proliferation and host defense [1], but excess ROS beyond the antioxidant systems of the organism give rise to oxidative stress [2]. With the development of free radical research over the past 60 years, it has become widely recognised that excessive and chronic oxidative stress is implicated in various pathological conditions such as cardiovascular diseases, cancer, neurodegenerative diseases, diabetes, and aging [3]. Therefore, maintaining the oxidation-reduction (redox) balance within an optimal range is crucial for the survival and health of an organism. To optimise the redox balance, it is necessary to identify lifestyle and biological factors that determine the levels of oxidative stress at rest.

In human studies, lifestyle and biological variables positively related to resting oxidative stress include alcohol consumption [4], smoking [4, 5], obesity [5–7], and aging [8, 9], as well as disease development [3]. On the other hand, variables that are negatively related to resting oxidative stress include calorie restriction [10], intake of antioxidants from food [5], antioxidant supplementation (e.g., vitamin C and vitamin E) [10], and anti-inflammatory medication [11]. In addition to these variables, gender has been suggested to be associated with resting oxidative stress, although no consensus has yet been reached [4, 5]. As such, many lifestyle and biological variables have been reported to potentially influence the regulation of resting oxidative stress; more importantly, the degree of influence of each variable on resting oxidative stress should be assessed comprehensively rather than separately. Indeed, several previous studies have explored the determinants of resting oxidative stress using multiple lifestyle and biological variables, such as age, sex, smoking status, and biochemical parameters [4–7, 12–14]. However, only one previous study has explored variables related to physical fitness, which are thought to be negatively related to oxidative stress [4]. In addition, as this previous study used broadly categorised exercise frequency as an independent variable, it is not clear whether objectively measured physical fitness can be a determinant of resting oxidative stress.

Therefore, the purpose of this study was to identify the lifestyle and biological variables that determine resting oxidative stress, including objectively measured physical fitness. For this purpose, two widely used and relatively reliable markers of oxidative stress [15, 16], protein carbonyl (PC) and F_2_-isoprostane (F_2_-IsoP), were measured spectrophotometrically using plasma samples from 873 participants of the WASEDA'S Health Study (Waseda Alumni's Sports, Exercise, Daily Activity, Sedentariness, and Health Study). In addition, thiobarbituric acid reactive substances (TBARS), which are now often criticised [17], were included in the analysis for comparison with data from previous studies. Along with these three markers of oxidative stress, a wide range of surveys and measurements were conducted, including objectively measured physical fitness, sex, age, anthropometric index, lifestyle-related parameters, medication and antioxidant supplementation status, biochemical parameters, and nutritional intake status. Through these measurements, the present study was aimed at providing evidence for establishing preventive strategies to avoid chronic oxidative stress.

## 2. Materials and Methods

### 2.1. Participants

This cross-sectional study used baseline data from the WASEDA'S Health Study. The WASEDA'S Health Study is a cohort study that investigates the relationship between health outcomes and sports, exercise, physical activity, and sedentary behaviour among alumni of Waseda University and their spouses aged 40 years or older.

The WASEDA'S Health Study consisted of four cohorts (Cohorts A-D). Participants in Cohort A completed an internet-based self-administered questionnaire on physical activity and health outcomes. Participants in Cohort B were asked to complete a self-administered questionnaire, as well as utilise an accelerometer to measure physical activity and sedentary time. Participants in Cohort C completed several medical tests in addition to the items in Cohort B. Participants in Cohort D performed a physical fitness test and detailed medical tests in addition to the items in Cohort C. Participants in the present study reported here were participants from Cohort D of the WASEDA'S Health Study.

**Figure 1:**
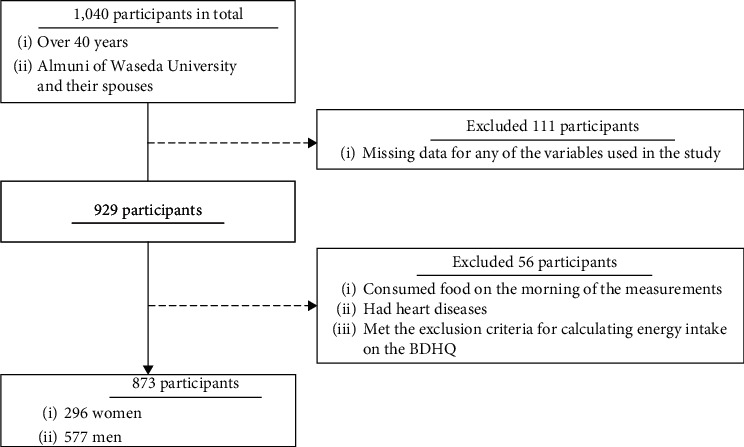
Selection and exclusion processes of the participants in the study.

The study included 1040 participants who took part in Cohort D in the WASEDA'S Health Study between March 2015 and July 2018. To perform an appropriate analysis, several inclusion and exclusion criteria were established in this study. First, those with missing data for any of the variables used in the study were excluded (*n* = 111). Second, those who consumed food on the morning of the measurement (*n* = 7), those who had heart diseases (*n* = 41), and those who met the exclusion criteria for calculating energy intake on the brief-type self-administered dietary history questionnaire (BDHQ) (*n* = 8) were excluded from the analysis. As a result of the exclusion and inclusion criteria, 873 individuals (296 women and 577 men) were ultimately included in the analysis of this study (Figure 1).

The participants were briefed on the study and signed an informed consent form prior to the baseline survey. This study was approved by the Research Ethics Committee of Waseda University (approval number: 2014-G002). The study was conducted in accordance with the Declaration of Helsinki (1964).

### 2.2. Questionnaire Survey

Self-administered questionnaires were used to determine the following: sex (man or woman), age (in years), exercise habits (whether or not they exercise regularly), smoking habits (current, former, and nonsmoking), frequency of drinking (less than once a week, 1–2 times a week, 3–4 times a week, and more than 5 times a week), antioxidant supplementation status (yes/no), and anti-inflammatory medication status (yes/no). BDHQ was also used to investigate energy intake (kcal/day), vitamin A (calculated as retinol activity equivalent) (*μ*g RAE/day), vitamin C (mg/day), *α*-tocopherol (mg/day), and *β*-carotene (calculated as *β*-carotene equivalent) (*μ*g/day). The BDHQ was assessed for validity in previous studies [18, 19].

### 2.3. Measurement of Physical Characteristics

Height (cm) and weight (kg) were measured using a stature meter (YHS-200D, YAGAMI Inc., Nagoya, Japan) and an anthropometer (MC-980A, Tanita, Tokyo, Japan). Weight measurements were taken with light clothing and with shoes off. Body mass index (BMI) (kg/m^2^) was calculated from height and weight measurements. Body fat percentage was measured using bioelectrical impedance analysis (MC-980A, Tanita, Tokyo, Japan). The lean body mass (LBM) (kg) was calculated from the weight and body fat percentage. Visceral fat area (VFA) (cm^2^) was measured at the umbilicus level, using magnetic resonance imaging (MRI) (SIGNA Premier, GE Healthcare, Waukesha, WI, USA), as described in a previous report [20].

### 2.4. Physical Fitness Tests

Cardiorespiratory fitness (CRF) (mL/kg/min) was measured using a bicycle ergometer (828E, Monarch, Stockholm, Sweden), and after 3 min of resting electrocardiogram measurements (ML-9000 and MXL-1000, Fukuda Denshi, Tokyo, Japan), men and women started at 30 W and 15 W, respectively, and the load was gradually increased by 15 W per minute until exhaustion. During each measurement, the oxygen and carbon dioxide concentrations in the exhaled gas were measured by the breath-by-breath method using an exhaled gas analyser with a mask for breath gas analysis (AE310S and AE100i, Minato Medical Science, Osaka, Japan). A blood pressure measurement cuff was wrapped around the left upper arm, and blood pressure (mmHg) was measured using an automatic sphygmomanometer for exercise testing (Tango M2, SunTech Medical, Morrisville, NC, USA). Heart rate (bpm) and blood pressure before and during exercise were used as the safety controls. The endpoint of the exercise stress test was a plateau in oxygen uptake or until the heart rate reached approximately 90% of the predicted maximum age-specific heart rate. However, they stopped the exercise test if they reached a rating of perceived exertion (RPE) of 18 or higher, if they reported an inability to continue exercising, or if their systolic blood pressure reached 250 mmHg. Following the exercise period, the participants took a 1 min active recovery and 2 minutes of seated rest to complete the exercise test. The highest average oxygen uptake per 30 s during exercise was set as the $\dot{\text{V}}{\text{O}}_{2}$ peak. The leg extension power (W) was measured five times repeatedly using a recumbent leg press (Anaeropress 3500, Combi, Tokyo, Japan), and the maximum value was adopted.

### 2.5. Blood Sampling and Measurement of Oxidative Stress

The participants were instructed to fast from the night before blood collection. Venous blood was collected from the forearm vein after checking for breakfast skipping at the time of blood collection. Blood was collected into a blood collection tube (Terumo Inc., Tokyo, Japan) with an anticoagulant (Heparin Na or EDTA-2Na) or a tube without an anticoagulant and centrifuged at 4°C at 3000 rpm for 10 min using a centrifuge (Model 5911, Kubota, Tokyo, Japan). The supernatant was transferred to microtubes, and both plasma and serum samples were stored in a freezer at -80°C until analysis. Serum samples were analysed for lipid metabolism (total cholesterol (total-C, in mg/dL), HDL cholesterol (HDL-C, in mg/dL), LDL cholesterol (LDL-C, in mg/dL), triglycerides (TG, in mg/dL)), and plasma samples were analysed for glucose metabolism (fasting blood glucose (glucose, in mg/dL) and haemoglobin A1c (HbA1c, in %)). All analyses of these items were conducted by an external institution (BML Inc., Tokyo, Japan).

Oxidative stress markers in plasma were measured using commercially available protein carbonyl colorimetric assay kits, 8-isoprostane ELISA kits, and TBARS assay kits (Cayman Chemical, Ann Arbor, MI, USA). Oxidative stress measurements were performed between March and August 2020, and all results were calculated based on standard curves using standard solutions. The intra- and interassay CVs of each biomarker were 8.8% and 12.0% for PC (nmol/mg protein), 6.4% and 8.0% for F_2_-IsoP (pg/mL), and 8.8% and 9.5% for TBARS (*μ*M), respectively, generally meeting the technical criteria proposed in a previous study [15].

### 2.6. Statistical Analysis

First, participants were classified by gender, and the physical characteristics and oxidative stress levels of each group were compared. The participants were also classified into tertiles for each oxidative stress marker, and the physical characteristics of each group were compared. For descriptive data, continuous variables are presented as mean ± standard deviation, and categorical variables are presented as the number of persons and percentages. Three multiple regression models were developed to identify the determinants of each oxidative stress marker, and the standard partial regression coefficients and 95% confidence intervals for the independent variables were calculated.

The dependent variables in each model were PC, F_2_-IsoP, and TBARS. The candidate independent variables in each model were age (years), BMI (kg/m^2^), LBM (kg), VFA (cm^2^), exercise habits (yes or no), smoking status (yes or no), drinking status (yes or no), antioxidant supplementation status (yes or no), anti-inflammatory medication status (yes or no), leg extension power (W), CRF (mL/kg/min), total cholesterol (mg/dL), HDL-C (mg/dL), LDL-C (mg/dL), TG (mg/dL), glucose (mg/dL), HbA1c (%), energy intake (kcal/day), vitamin A (*μ*g RAE/day), vitamin C (mg/day), *α*-tocopherol (mg/day), and *β*-carotene equivalent (*μ*g/day). The stepwise method was used to develop the model, and the forward selection method was used as a variable selection method. Before using the stepwise method, the variance inflation factor for each variable was calculated to avoid multicollinearity and to ensure that the VIF was less than 10. The probability for being stepwise was set to 0.10, for the *P* value at the time of input, and 0.15, for the *P* value at the time of removal. Although VFA was used as a measure of obesity in this study, the analyses were conducted using body fat percentage instead of VFA as a sensitivity analysis. Since this study was exploratory, the increase in alpha error due to multiple comparisons was not considered, and statistical significance was expressed when the two-sided *P* value was less than 5%. All statistical analyses were performed using SPSS Statistics version 26 (IBM Corporation, Chicago, IL, USA).

## 3. Results

### 3.1. Characteristics of the Participants

The characteristics of all participants and participants by sex are presented in Table 1. The overall number of participants in this study was 873, of which 296 were women and 577 were men.

### 3.2. Resting Oxidative Stress Levels of the Participants

The resting oxidative stress levels of the participants are presented in Table 2. Participant characteristics by tertiles for each oxidative stress marker are shown in Tables 3–5.

### 3.3. Stepwise Multiple Regression Analysis of Each Oxidative Stress Marker in All Participants

The results of the stepwise multiple regression analysis of each oxidative stress marker are presented in Table 6. With PC as the dependent variable, age (*β* = −0.11, *P* = 0.002), leg extension power (*β* = −0.12, *P* = 0.008), BMI (*β* = 0.12, *P* = 0.004), and HDL-C (*β* = 0.08, *P* = 0.040) were included in the regression model (adjusted *R*^2^ = 0.018). In the F_2_-IsoP, smoking status (*β* = 0.07, *P* = 0.060), BMI (*β* = 0.07, *P* = 0.054), and HbA1c (*β* = −0.06, *P* = 0.089) were included in the regression model (adjusted *R*^2^ = 0.006). In TBARS, glucose (*β* = 0.18, *P* < 0.001), CRF (*β* = 0.16, *P* < 0.001), age (*β* = 0.15, *P* < 0.001), TG (*β* = 0.11, *P* = 0.001), antioxidant supplementation (*β* = 0.10, *P* = 0.002), and HbA1c (*β* = −0.13, *P* = 0.004) were included in the regression model (adjusted *R*^2^ = 0.071).

### 3.4. Stepwise Multiple Regression Analysis of Each Oxidative Stress Marker in Men and Women

The results of the multiple regression analysis of each oxidative stress marker in women are presented in Table 7. With PC as the dependent variable, anti-inflammatory medication (*β* = −0.12, *P* = 0.044) was included in the regression model (adjusted *R*^2^ = 0.010). In the F_2_-IsoP, smoking status (*β* = 0.16, *P* = 0.005), BMI (*β* = 0.14, *P* = 0.016), antioxidant supplementation (*β* = −0.11, *P* = 0.050), and energy intake (*β* = 0.10, *P* = 0.078) were included in the regression model (adjusted *R*^2^ = 0.052). In TBARS, *α*-tocopherol (*β* = 0.27, *P* = 0.003), *β*-carotene (*β* = −0.24, *P* = 0.008), CRF (*β* = 0.23, *P* < 0.001), age (*β* = 0.25, *P* < 0.001), HbA1c (*β* = −0.14, *P* = 0.033), anti-inflammatory medication (*β* = 0.13, *P* = 0.017), and leg extension power (*β* = −0.16, *P* = 0.006) were included in the regression model (adjusted *R*^2^ = 0.090). The results of the multiple regression analysis of each oxidative stress marker in men are presented in Table 8. With PC as the dependent variable, anti-inflammatory medication (*β* = 0.11, *P* = 0.006), age (*β* = −0.12, *P* = 0.006), and visceral fat area (*β* = 0.08, *P* = 0.046) were included in the regression model (adjusted *R*^2^ = 0.024). In the F_2_-IsoP, none of the variables were included in the regression model (adjusted *R*^2^ = 0.033). Antioxidant supplementation (*β* = 0.12, *P* = 0.003), TG (*β* = 0.13, *P* = 0.002), glucose (*β* = 0.23, *P* < 0.001), VFA (*β* = −0.11, *P* = 0.011), and HbA1c (*β* = −0.14, *P* = 0.019) were included in the regression model (adjusted *R*^2^ = 0.056).

## 4. Discussion

The present study explored lifestyle and biological variables that determine resting oxidative stress levels, including objectively measured physical fitness, in middle-aged and elderly men and women. As a result, several determinants were identified which may be related to the redox regulation of each oxidative stress marker. However, the variables selected in this study, such as age, anthropometric index, lifestyle-related parameters, medication and supplementation status, objectively measured physical fitness, biochemical parameters, and nutritional intake status, were very weakly associated with oxidative stress.

Although moderate oxidative stress is essential for homeostasis, excessive and chronic oxidative stress has been shown to be partially involved in the development and progression of various diseases and biological aging [3]. Thus, maintaining oxidative stress within an optimal range is expected to contribute to disease prevention and the extension of healthy life expectancy [21]. In this context, identifying and weighting the lifestyle and biological variables associated with resting oxidative stress would be important in prioritising future redox control strategies. Therefore, the present study attempted to identify and weigh the determinants of resting oxidative stress using data from a variety of variables that include objectively measured physical fitness.

The results of the present study revealed that the regression equations of all participants were significant (PC: *P* = 0.001, F_2_-IsoP: *P* = 0.036, and TBARS: *P* = 0.004, respectively), but the regression coefficients *R*^2^ were small (PC = 0.018, F_2_‐IsoP = 0.006, and TBARS = 0.071), revealing low predictive accuracy (Table 6). Consistent with the results of all participants, the subgroup analyses conducted separately for men and women obtained roughly similar results (Tables 7 and 8). Focusing on the previous studies, to the best of our knowledge, there are seven papers that have examined the determinants of resting oxidative stress [4–7, 12–14]. In summary, oxidative stress markers such as TBARS, F_2_-IsoP, 4-hydroxynonenal bound to thiol proteins (4HNE-P), 8-hydroxydeoxyguanosine, and hydrogen peroxidase production have been measured using biological samples such as plasma, erythrocyte and leukocyte lysates, and urine. In addition, the age of the participants ranged from 19 to 88 years, and the number of participants varied widely from 61 to 2828. Furthermore, age, sex, anthropometric index, lifestyle-related parameters (including exercise habits), medication and supplementation status, biochemical parameters, and nutritional intake status were included as independent variables. In these previous studies, the adjusted *R*^2^ of the regression models ranged from 0.024 to 0.569 [4–7, 12–14]. Compared to these results, the prediction accuracy of each regression model in this study appears to be relatively low (adjusted *R*^2^ = 0.006–0.071).

Of the previous studies that have examined the determinants of oxidative stress, only three have used plasma as a biological sample [5, 12, 14]. Trevisan et al. examined the determinants of plasma TBARS levels in 1797 men and women aged 35-79 years using multiple regression analysis and reported that gender, plasma glucose, TG, and HDL-C were each associated with the TBARS level [12]. In addition, a study of 298 healthy men and women aged 19-78 years by Block et al. suggested that sex, race, BMI, smoking status, plasma ascorbic acid, *γ*-tocopherol, *β*-carotene, C-reactive protein, and total-C were determinants of plasma TBARS and/or F_2_-IsoP levels [5]. Furthermore, a previous study of 61 men and women with heart failure by Asselin et al. showed the involvement of BMI, medication status, blood pressure, blood linoleic acid, HDL-C, total bilirubin, reduced glutathione/oxidized glutathione ratio (GSH/GSSG ratio), glucose, alkaline phosphatase, and myeloperoxidase as determinants of plasma 4HNE-P and TBARS levels [14]. Taken together, this limited evidence suggests that variables such as sex, race, BMI, smoking status, blood pressure, medication status, and biochemical parameters can partially explain resting oxidative stress levels. In addition to these variables, age, leg extension power, CRF, and antioxidant supplementation status were significantly associated with oxidative stress markers in the present study (Table 6). However, the variables selected in the present study can only explain less than 10% of oxidative stress at rest, even though they are partially consistent with those of previous studies [5, 12, 14].

The discrepancy in prediction accuracy between previous studies and the present study may be due to differences in the study conditions, such as the target population, the oxidative stress markers selected (i.e., PC, F_2_-IsoP, TBARS, 4HNE-P, and hydrogen peroxide production), and the methods of measuring oxidative stress (e.g., spectrophotometry and high-performance liquid chromatography analysis). However, it is difficult to identify a clear reason for the difference in prediction accuracy. Therefore, further research into the determinants of resting oxidative stress is necessitated.

## 5. Conclusions

This study identified several lifestyle and biological variables that determine resting oxidative stress in middle-aged and elderly men and women and the contribution of independent variables to each oxidative stress marker. However, the present study showed that anthropometric index, lifestyle-related parameters, medication and supplementation status, objectively measured physical fitness, biochemical parameters, and nutritional intake status explain less than 10% of oxidative stress levels at rest.

## Figures and Tables

**Table 1:** Characteristics of the participants by gender.

	All (*n* = 873)	Women (*n* = 296)	Men (*n* = 577)
*Age and anthropometric index*			
Age (years)	55 ± 10	52 ± 9	56 ± 10
BMI (kg/m^2^)	22.9 ± 3.1	21.3 ± 2.7	23.7 ± 3.0
LBM (kg)	48.8 ± 9.0	38.4 ± 3.1	54.2 ± 5.4
VFA (cm^2^)	75.2 ± 42.3	51.0 ± 29.5	87.6 ± 42.5
*Lifestyle-related parameters*			
Exercise habits (*n*, %)	300 (34.4)	114 (38.5)	186 (32.2)
Smokers (*n*, %)	54 (6.2)	7 (2.4)	47 (8.1)
Nondrinker (*n*, %)	177 (20.4)	102 (24.3)	75 (18.4)
*Medication and supplementation status*			
Antioxidant supplementation (*n*, %)	116 (13.3)	49 (16.6)	67 (11.6)
Anti-inflammatory medication (*n*, %)	31 (3.6)	14 (4.7)	17 (2.9)
*Physical fitness*			
Leg extension power (W)	1083 ± 425	701 ± 222	1279 ± 367
CRF (mL/kg/min)	28.8 ± 6.3	26.0 ± 5.1	30.2 ± 6.3
*Biochemical parameters*			
Total-C (mg/dL)	211 ± 36	214 ± 38	209 ± 35
HDL-C (mg/dL)	66 ± 17	74 ± 16	62 ± 16
LDL-C (mg/dL)	121 ± 31	118 ± 33	122 ± 30
TG (mg/dL)	99 ± 79	75 ± 42	111 ± 90
Glucose (mg/dL)	96 ± 14	90 ± 9	98 ± 15
HbA1c (%)	5.4 ± 0.4	5.3 ± 0.4	5.5 ± 0.5
*Nutritional intake status*			
Energy intake (kcal/day)	1973 ± 526	1758 ± 448	2082 ± 529
Vitamin A (*μ*g RAE/day)	930 ± 496	888 ± 457	952 ± 514
Vitamin C (mg/day)	131 ± 63	135 ± 63	129 ± 62
*α*-Tocopherol (mg/day)	8.5 ± 3.0	8.3 ± 3.0	8.6 ± 3.0
*β*-Carotene (*μ*g/day)	4467 ± 2852	5065 ± 3046	4159 ± 2699

Data for continuous variables are presented as the mean ± standard deviation (SD), and data for categorical variables are presented as the number of persons (*n*) and percentage (%) of applicable persons. BMI: body mass index; LBM: lean body mass; VFA: visceral fat area; CRF: cardiorespiratory fitness; total-C: total cholesterol; HDL-C: HDL cholesterol; LDL-C: low-density lipoprotein cholesterol; TG: triglycerides; glucose: fasting blood glucose; HbA1c: haemoglobin A1c; vitamin A: retinol activity equivalent; *β*-carotene: *β*-carotene equivalent.

**Table 2:** Resting oxidative stress levels of the participants.

	All (*n* = 873)	Women (*n* = 296)	Men (*n* = 577)
PC (nmol/mg protein)	1.8 ± 0.9	1.9 ± 1.0	1.7 ± 0.8
F_2_-IsoP (pg/mL)	38.8 ± 31.6	41.1 ± 32.7	37.6 ± 31.0
TBARS (*μ*M)	2.5 ± 0.6	2.3 ± 0.6	2.6 ± 0.6

Data are presented as means ± standard deviation (SD). PC: protein carbonyl; F_2_-IsoP: F_2_-isoprostane; TBARS: thiobarbituric acid reactive substances.

**Table 3:** Characteristics of the participants by protein carbonyl tertiles.

	T1 (*n* = 291)	T2 (*n* = 291)	T3 (*n* = 291)
*Age and anthropometric parameters*			
Age (years)	56 ± 10	55 ± 9	53 ± 9
BMI (kg/m^2^)	22.9 ± 3.2	22.8 ± 3.0	23.0 ± 3.2
LBM (kg)	49.0 ± 8.5	48.9 ± 8.9	48.6 ± 9.4
VFA (cm^2^)	73.4 ± 43.4	75.7 ± 42.7	76.5 ± 40.9
*Lifestyle-related parameters*			
Exercise habits (*n*, %)	197 (67.7)	191 (65.6)	185 (63.6)
Smokers (*n*, %)	18 (6.2)	19 (6.5)	17 (5.8)
Nondrinker (*n*, %)	55 (18.9)	67 (23.0)	55 (18.9)
*Medication and supplementation status*			
Antioxidant supplementation (*n*, %)	43 (14.8)	34 (11.7)	39 (13.4)
Anti-inflammatory medication (*n*, %)	10 (3.4)	11 (3.8)	10 (3.4)
*Physical fitness*			
Leg extension power (W)	1093 ± 419	1096 ± 437	1059 ± 419
CRF (mL/kg/min)	29.2 ± 6.7	28.6 ± 6.1	28.6 ± 5.9
*Biochemical parameters*			
Total-C (mg/dL)	210 ± 36	210 ± 35	212 ± 38
HDL-C (mg/dL)	66 ± 17	64 ± 17	68 ± 17
LDL-C (mg/dL)	119 ± 33	122 ± 30	121 ± 32
TG (mg/dL)	99 ± 68	101 ± 102	96 ± 61
Glucose (mg/dL)	96 ± 13	95 ± 13	96 ± 14
HbA1c (%)	5.4 ± 0.5	5.4 ± 0.4	5.4 ± 0.4
*Nutritional intake status*			
Energy intake (kcal/day)	1997 ± 553	1955 ± 503	1967 ± 522
Vitamin A (*μ*g RAE/day)	899 ± 478	965 ± 490	926 ± 518
Vitamin C (mg/day)	132 ± 62	133 ± 63	127 ± 62
*α*-Tocopherol (mg/day)	8.6 ± 3.1	8.4 ± 2.9	8.4 ± 2.0
*β*-Carotene (*μ*g/day)	4483 ± 2827	4675 ± 3009	4242 ± 2706

Data for continuous variables are presented as the mean ± standard deviation (SD), and data for categorical variables are presented as the number of persons (*n*) and percentage (%). BMI: body mass index; LBM: lean body mass; VFA: visceral fat area; CRF: cardiorespiratory fitness; total-C: total cholesterol; HDL-C: HDL cholesterol; LDL-C: low-density lipoprotein cholesterol; TG: triglycerides; glucose: fasting blood glucose; HbA1c: haemoglobin A1c; vitamin A: retinol activity equivalent; *β*-carotene: *β*-carotene equivalent.

**Table 4:** Characteristics of the participants by F_2_-isoprostane tertiles.

	T1 (*n* = 291)	T2 (*n* = 291)	T3 (*n* = 291)
*Age and anthropometric parameters*			
Age (years)	54 ± 9	55 ± 10	54 ± 10
BMI (kg/m^2^)	22.6 ± 2.9	22.9 ± 2.8	23.1 ± 3.6
LBM (kg)	48.8 ± 8.7	49.4 ± 8.7	48.3 ± 9.3
VFA (cm^2^)	71.7 ± 37.3	79.4 ± 45.0	74.4 ± 43.9
*Lifestyle-related parameters*			
Exercise habits (*n*, %)	190 (65.3)	194 (66.7)	189 (64.9)
Smokers (*n*, %)	21 (7.2)	14 (4.8)	19 (6.5)
Nondrinker (*n*, %)	65 (22.3)	49 (16.8)	63 (21.6)
*Medication and supplementation status*			
Antioxidant supplementation (*n*, %)	47 (16.2)	33 (11.3)	36 (12.4)
Anti-inflammatory medication (*n*, %)	16 (5.5)	4 (1.4)	11 (3.8)
*Physical fitness*			
Leg extension power (W)	1090 ± 434	1090 ± 413	1068 ± 428
CRF (mL/kg/min)	29.4 ± 6.1	28.4 ± 6.4	28.5 ± 6.3
*Biochemical parameters*			
Total-C (mg/dL)	211 ± 37	209 ± 34	212 ± 38
HDL-C (mg/dL)	67 ± 18	65 ± 17	67 ± 17
LDL-C (mg/dL)	121 ± 34	120 ± 29	121 ± 32
TG (mg/dL)	95 ± 58	102 ± 101	99 ± 72
Glucose (mg/dL)	96 ± 12	96 ± 16	95 ± 12
HbA1c (%)	5.5 ± 0.4	5.4 ± 0.5	5.4 ± 0.4
*Nutritional intake status*			
Energy intake (kcal/day)	1967 ± 536	1980 ± 520	1971 ± 524
Vitamin A (*μ*g RAE/day)	133 ± 60	127 ± 61	132 ± 67
Vitamin C (mg/day)	8.6 ± 3.1	8.2 ± 2.8	8.6 ± 3.0
*α*-Tocopherol (mg/day)	4721 ± 3041	4161 ± 2606	4518 ± 2874
*β*-Carotene (*μ*g/day)	949 ± 460	910 ± 515	932 ± 512

Data for continuous variables are presented as the mean ± standard deviation (SD), and data for categorical variables are presented as the number of persons (*n*) and percentage (%). BMI: body mass index; LBM: lean body mass; VFA: visceral fat area; CRF: cardiorespiratory fitness; total-C: total cholesterol; HDL-C: HDL cholesterol; LDL-C: low-density lipoprotein cholesterol; TG: triglycerides; glucose: fasting blood glucose; HbA1c: haemoglobin A1c; vitamin A: retinol activity equivalent; *β*-carotene: *β*-carotene equivalent.

**Table 5:** Characteristics of the participants by thiobarbituric acid reactive substance tertiles.

	T1 (*n* = 291)	T2 (*n* = 291)	T3 (*n* = 291)
*Age and anthropometric parameters*			
Age (years)	53 ± 9	55 ± 10	55 ± 9
BMI (kg/m^2^)	22.7 ± 3.3	22.9 ± 3.2	23.0 ± 2.9
LBM (kg)	47.2 ± 9.0	48.8 ± 9.0	50.5 ± 8.3
VFA (cm^2^)	72.2 ± 42.5	77.5 ± 44.4	75.9 ± 40.0
*Lifestyle-related parameters*			
Exercise habits (*n*, %)	182 (62.3)	197 (67.9)	194 (66.7)
Smokers (*n*, %)	16 (5.5)	16 (5.5)	22 (7.6)
Nondrinker (%)	70 (24.0)	58 (20.0)	49 (16.8)
*Medication and supplementation status*			
Antioxidant supplementation (*n*, %)	30 (10.3)	37 (12.8)	49 (16.8)
Anti-inflammatory medication (*n*, %)	7 (2.4)	13 (4.5)	11 (3.8)
*Physical fitness*			
Leg extension power (W)	1028 ± 406	1068 ± 438	1152 ± 423
CRF (mL/kg/min)	27.6 ± 5.7	28.9 ± 6.3	29.8 ± 6.5
*Biochemical parameters*			
Total-C (mg/dL)	205 ± 36	215 ± 36	211 ± 36
HDL-C (mg/dL)	66 ± 17	67 ± 17	65 ± 17
LDL-C (mg/dL)	117 ± 31	125 ± 32	121 ± 31
TG (mg/dL)	89 ± 54	101 ± 67	106 ± 106
Glucose (mg/dL)	93 ± 8.6	96 ± 13	98 ± 18
HbA1c (%)	5.4 ± 0.3	5.4 ± 0.5	5.4 ± 0.5
*Nutritional intake status*			
Energy intake (kcal/day)	1922 ± 515	1973 ± 525	2024 ± 535
Vitamin A (*μ*g RAE/day)	129 ± 64	132 ± 63	131 ± 61
Vitamin C (mg/day)	8.3 ± 3.0	8.5 ± 2.9	8.6 ± 3.1
*α*-Tocopherol (mg/day)	4495 ± 2891	4482 ± 2915	4422 ± 2757
*β*-Carotene (*μ*g/day)	937 ± 518	907 ± 472	947 ± 498

Data for continuous variables are presented as the mean ± standard deviation (SD), and data for categorical variables are presented as the number of persons (*n*) and percentage (%). BMI: body mass index; LBM: lean body mass; VFA: visceral fat area; CRF: cardiorespiratory fitness; total-C: total cholesterol; HDL-C: HDL cholesterol; LDL-C: low-density lipoprotein cholesterol; TG: triglycerides; glucose: fasting blood glucose; HbA1c: haemoglobin A1c; vitamin A: retinol activity equivalent; *β*-carotene: *β*-carotene equivalent.

**Table 6:** Stepwise multiple regression analysis of each oxidative stress marker in all participants.

	Partial regression coefficient	95% confidence interval	Standard partial regression coefficient	VIF	*P* value
*PC* ^∗^^1^					
Intercept	1.518	0.795–2.242			<0.001
Age (years)	-0.010	-0.016–−0.004	-0.11	1.10	0.002
Leg extension power (W)	0.000022	0.000–0.000	-0.12	1.38	0.008
BMI (kg/m^2^)	0.034	0.011–−0.057	0.12	1.55	0.004
HDL-C (mg/dL)	0.004	0.000–0.008	0.08	1.34	0.040
*F_2_-IsoP* ^∗^^2^					
Intercept	37.208	5.622–68.794			0.021
Smokers	3.403	-0.145–6.952	0.07	1.07	0.060
BMI (kg/m^2^)	0.690	-0.013–1.393	0.07	1.09	0.054
HbA1c (%)	-4.185	-9.009–0.640	-0.06	1.05	0.089
*TBARS* ^∗^^3^					
Intercept	1.634	1.055–2.213			<0.001
Glucose (mg/dL)	0.008	0.004–0.012	0.18	1.98	<0.001
CRF (mL/kg/min)	0.016	0.009–0.023	0.16	1.15	<0.001
Age (years)	0.009	0.005–0.014	0.15	1.29	<0.001
TG (mg/dL)	0.001	0.000–0.001	0.11	1.05	0.001
Antioxidant supplementation	0.186	0.070–0.303	0.10	1.01	0.002
HbA1c (%)	0.183	-0.308–−0.057	-0.13	1.99	0.004

VIF: variance inflation factor; PC: protein carbonyl; F_2_-IsoP: F_2_-isoprostane; TBARS: thiobarbituric acid reactive substances; LBM: lean body mass; BMI: body mass index; HDL-C: high-density lipoprotein cholesterol; HbA1c: haemoglobin A1c; glucose: fasting blood glucose; CRF: cardiorespiratory fitness; TG: triglycerides. ^∗1^*R*^2^ = 0.022, adjusted *R*^2^ = 0.018; ^∗2^*R*^2^ = 0.010, adjusted *R*^2^ = 0.006; ^∗3^*R*^2^ = 0.077, adjusted *R*^2^ = 0.071.

**Table 7:** Stepwise multiple regression analysis of each oxidative stress marker in women.

	Partial regression coefficient	95% confidence interval	Standard partial regression coefficient	VIF	*P* value
*PC* ^∗^^1^					
Intercept	1.905	1.789–2.021			<0.001
Anti-inflammatory medication	-0.547	-1.080–0.015	-0.12	1.00	0.044
*F_2_-IsoP* ^∗^^2^					
Intercept	-38.144	-80.099–3.810			0.075
Smokers	11.447	3.408–19.486	0.16	1.03	0.005
BMI (kg/m^2^)	1.690	0.312–3.068	0.14	1.04	0.016
Antioxidant supplementation	-9.859	-19.724–−0.006	-0.11	1.01	0.050
Energy intake (kcal/day)	0.007	-0.001–−0.016	0.10	1.01	0.078
*TBARS* ^∗^^3^					
Intercept	2.058	0.967–3.149			<0.001
*α*-Tocopherol (mg/day)	0.054	0.019–0.090	0.27	2.71	0.003
*β*-Carotene (*μ*g/day)	-4.556	0.000–0.000	-0.24	2.64	0.008
CRF (mL/kg/min)	0.026	0.012–0.039	0.23	1.23	<0.001
Age (years)	0.017	0.008–0.027	0.25	1.65	<0.001
HbA1c (%)	-0.231	-0.443–−0.019	-0.14	1.41	0.033
Anti-inflammatory medication	0.362	0.065–0.659	0.13	1.01	0.017
Leg extension power (W)	0.00004	-0.001–0.000	-0.16	1.11	0.006

VIF: variance inflation factor; PC: protein carbonyl; F_2_-IsoP: F_2_-isoprostane; TBARS: thiobarbituric acid reactive substances; BMI: body mass index; CRF: cardiorespiratory fitness; HbA1c: haemoglobin A1c. ^∗1^*R*^2^ = 0.014, adjusted *R*^2^ = 0.010; ^∗2^*R*^2^ = 0.065, adjusted *R*^2^ = 0.052; ^∗3^*R*^2^ = 0.158, adjusted *R*^2^ = 0.090.

**Table 8:** Stepwise multiple regression analysis of each oxidative stress marker in men.

	Partial regression coefficient	95% confidence interval	Standard partial regression coefficient	VIF	*P* value
*PC* ^∗^^1^					
Intercept	2.108	1.724–2.491			<0.001
Anti-inflammatory medication	0.556	0.161–0.003	0.11	1.00	0.006
Age (years)	-0.010	-0.016–0.121	-0.12	1.05	0.006
VFA (cm^2^)	0.002	0.000–−0.003	0.08	1.05	0.046
*F_2_-IsoP* ^∗^^2^					
—	—	—	—	—	—
*TBARS* ^∗^^3^					
Intercept	2.647	2.075–3.219			<0.001
Antioxidant supplementation	0.234	0.080–0.387	0.12	1.98	0.003
TG (mg/dL)	0.001	0.000–0.001	0.13	1.15	0.002
Glucose (mg/dL)	0.009	0.004–0.014	0.23	1.29	<0.001
VFA (cm^2^)	-0.002	-0.003–0.000	-0.11	1.05	0.011
HbA1c (%)	-0.180	-0.331–−0.030	-0.14	1.01	0.019

VIF: variance inflation factor; PC: protein carbonyl; F_2_-IsoP: F_2_-isoprostane; TBARS: thiobarbituric acid reactive substances; VFA: visceral fat area; TG: triglycerides; glucose: fasting blood glucose; HbA1c: haemoglobin A1c. ^∗1^*R*^2^ = 0.029, adjusted *R*^2^ = 0.024; ^∗2^*R*^2^ = 0.043, adjusted *R*^2^ = 0.033; ^∗3^*R*^2^ = 0.064, adjusted *R*^2^ = 0.056.

## Data Availability

The measurement data of all participants used to support the findings of this study are available from the corresponding author upon request.
